# How to co-create context-sensitive analyses of health service experiences: proposed guidelines for contextualist collaborative thematic analyses with co-researchers

**DOI:** 10.1080/17482631.2026.2673592

**Published:** 2026-05-15

**Authors:** Nina K. H. Bahl, Morten Brodahl, Hilde E. Nafstad, Rolv M. Blakar

**Affiliations:** a Department of Research and Development, Clinic of Substance Use and Addiction Medicine, St. Olavs University Hospital, Trondheim, Norway; b Research Centre for Substance Use Disorders and Mental Illness, Innlandet Hospital Trust, Divisjon Psykisk Helsevern, Brumunddal, Norway; c Department of Psychology, University of Oslo, Oslo, Norway

**Keywords:** User-involvement, collaborative analysis, cultural-ideological, health services research, context-sensitive, co-researcher

## Abstract

**Purpose:**

User involvement is a prominent requirement in health services research across international health service contexts. However, current limitations with user-involved analytical approaches restrict researchers' sensitization to service users' contexts in analysis, with detrimental consequences for rigor and validity. In this methods article, we propose a novel analytical approach for how to conduct contextualist collaborative thematic analyses with co-researchers, developed through processes of previous cultural-ideological and collaborative thematic analyses.

**Method:**

This method's article introduces a cultural-ideological framework, as well as analytical and epistemological positioning for a four-stage procedure for conducting cultural-ideological and collaborative thematic analysis with co-researchers: 1) Coding and development of initial themes, 2) Collaborative reviewing and contextual interpretation of themes, 3) Collaborative synthesis of themes, and 4) Final adjustment of themes and dissemination of findings.

**Results:**

We demonstrate how the proposed approach adds experiential and contextual depth and breadth in analyses and aligns with established validity dimensions.

**Conclusions:**

This article contributes to the scholarship on qualitative user-involved approaches by adding a context-sensitive approach aligned with evolving values and epistemology in health services research. While the approach presents several challenges, it offers valuable implications for rigorous and valid understandings of health service experiences across cultures.

## Introduction

User involvement has become an increasingly prominent requirement in health services research across international health service contexts (Lu et al., 2025; Mathieson et al., 2025; Pedersen et al., 2023). Over recent decades, service users have been recognized not only as sources of data but also as contributors to the research process, including the formulation of research questions, data interpretation, and dissemination of findings (Mathieson et al., 2025; Pedersen et al., 2023). These developments reflect broader shifts in values and epistemologies within health services research, moving away from positivist and consumerist orientations toward participatory and empowerment-oriented approaches emphasizing control, accountability, and situated knowledge (Boote et al., 2002; Levitt et al., 2025; Wisdom et al., 2012).

Collaborative analysis with co-researchers^1^ has been recognized as a particularly appropriate way of conducting user-involved research within health services research (Eggebø, 2020; Moltu et al., 2013; Pettersen et al., 2019; Shippee et al., 2015; Thurston et al., 2023). However, there are still quite a few detailed descriptions of how researchers and co-researchers can collaboratively conduct analyzes of servicer users' experiences together, particularly in a context-sensitive^2^ manner. As such, health services researchers lack structured guidelines and frameworks for ensuring rigorous and valid understandings of health service experiences across the world.

In this methods article, we propose a cultural-ideological framework of co-researchers, as well as a novel analytical approach for how to conduct contextualist collaborative thematic analyzes (TA) with co-researchers. Specifically, we propose guidelines for a four-stage, step-by-step procedure for conducting cultural-ideological and collaborative thematic analysis with co-researchers, designed to support context-sensitive and valid understandings of situated health service experiences. This approach has been developed through the processes of previous cultural-ideological analyzes (Bahl & Hagen, 2017; Bahl et al., 2021, 2025) and collaborative thematic analyzes of data from a national Norwegian qualitative health services research project: “Brukererfaringsundersøkelsen” (Bahl et al., 2019, 2022, 2023a, 2023b).

## Limitations with user-involved analytical approaches in health services research

Three interrelated limitations characterize current user-involved analytical approaches within health services research. First, systematic descriptions of collaborative analytical approaches remain scarce. While the value of user-involvement in research is well established, there are still relatively few detailed accounts of how researchers and co-researchers can collaboratively conduct analyzes of service users' experiences (Shippee et al., 2015). Second, the majority of approaches insufficiently reflect ongoing epistemological shifts in the field, continuing to rest on consumerist and market-driven assumptions, such as satisfaction and value for money, rather than political and empowerment-oriented models emphasizing service users' agency, control, and accountability (Boote et al., 2002). While positivist health services research is, of course, needed and can include user involvement in analysis, there remains a lack of analytical approaches aligned with these epistemological developments. Third, analyzes are largely confined to the individual level, with limited attention to the institutional and cultural-ideological contexts in which service–user experiences are embedded. While qualitative inquiries traditionally emphasize lived experience and sensitivity to participants' worlds and contexts (Gergen et al., 2015; Yardley, 2015), health service users and co-researchers, are still often treated as context-less individuals in analyzes of health service experiences.

Taken together, these limitations have important implications for the rigour and validity of qualitative health services research. In the absence of detailed guidelines, user involvement in analysis risks becoming confined to consultative or symbolic forms rather than functioning as a genuine analytical resource. Furthermore, analytical approaches that are misaligned with ongoing shifts in values and epistemologies or fail to integrate sensitivity to levels beyond the individual level risk generating understandings of health service-related phenomena that lack crucial contextual and experiential nuances. Consequently, findings may have limited relevance for both service providers and service users across the world.

To lay the foundations for an analytical approach aligned with ongoing epistemological shifts and established validity dimensions for qualitative research, we will now present a cultural-ideological framework of co-researchers' meaning systems.

## Materials and methods

### A cultural-ideological framework of co-researchers' meaning systems

All human experiences, and thus also service users' and co-researchers' experiences, are influenced by meaning systems^3^, values, and ideologies within a culture (Doise, 2011; Kim & Park, 2007; Kirmayer, 2025; Nafstad & Blakar, 2012; Valentim, 2011). As such, and most importantly, health service experiences do not occur in a vacuum; they are shaped by broader cultural and ideological contexts, spanning micro-, meso-, exo-, macro-, and chronosystem levels (Bronfenbrenner, 1994; Levitt et al., 2025; Nafstad & Blakar, 2012; Valentim, 2011).

Their micro-level meaning systems are thus embedded in macro-level meaning systems of ideas and beliefs shared within the Norwegian cultural-ideological context. As such, co-researchers meaning systems can provide access to a situated understanding of service users' experiences. Furthermore, co-researchers' meaning systems can function as “cultural-ideological keys” to understand other service users' situated experiences from the shared health services.

While individual co-researchers will have their own unique experiences of health services, including their user perspective in analysis, this allows for a deeper and broader understanding of situated service user experiences than would be possible from a researcher's perspective alone. Thus, collaborative analyzes with co-researchers—ideally several^4^—incorporating their meaning systems into the interpretative process, may ultimately lead to enhanced context-sensitive understandings within health services inquiries.

This may seem like an uncritical portrayal of co-researchers in research, and some may ask: “Will the co-researcher and their preconceptions not lead them to biased interpretations of the data?” And our answer is “yes”. However, just as reflexive thematic analysis (TA), which is the fundament for the current proposed approach, this analytical approach embraces researcher—and co-researcher—subjectivity (thus, rejecting positivist notions of researcher bias) as a resource for research, viewing qualitative analyzes as inherently subjective (Braun & Clarke, 2023). What is crucial for good analytical practice is not to code “accurately” or in a representative way, but to be reflexive and transparent about one's own perspective and how it may influence the findings. Furthermore, while lived or living experience constitutes a central analytical resource, it is not sufficient in itself for collaborative analysis. Co-researchers are required to possess basic analytical competencies, including familiarity with qualitative research ethics, reflexive practices, and principles of qualitative analysis.

Finally, for collaborative thematic analyzes with co-researchers to be systematic and context-sensitive, we need a structured analytical approach integrating a cultural-ideological framework in a procedural manner. Before we can introduce concrete guidelines for the proposed cultural-ideological approach, however, we have to outline its analytical and epistemological positioning.

## Analytical and epistemological positioning

TA is a widely applied analytical approach in health services research and is recognized as flexible, accessible, and relatively easy to learn (Braun & Clarke, 2014). These characteristics make it particularly appropriate as a foundation for research teams including members with varying levels of analytical experience, as is often the case in collaborative research. Collaborative thematic analyzes are conducted collaboratively if the goal is to increase reflexivity and interpretative depth (Braun & Clarke, 2019). Collaborative analyzes are, thus, undertaken as multiple perspectives can provide greater reflexivity and interpretative depth to the analysis.

Collaborative thematic analyzes with co-researchers, then, have the purpose of including the co-researcher perspective, to a) reach a deeper understanding of user experiences than we could achieve by a researchers' perspective alone, and b) obtain a more nuanced understanding of these experiences as well as the analysts' interpretations of these. As such, the proposed analytical approach positions itself with collaborative thematic analysis (Braun & Clarke, 2019) as well as collaborative qualitative data analysis (Richards & Hemphill, 2018), in its aim to utilize and integrate different analysts' perspectives for a deeper and broader analysis.

However, the proposed approach is also positioned within a cultural-ideological framework (Elder, 1974, 1980; Billig, 1997; Nafstad & Blakar, 2012), in its aim to co-construct context-sensitive analyzes with co-researchers. Thus, resonating with this framework, it takes a contextualist epistemological position, where reality is assumed to be “out there” and that access to reality is mediated by sociocultural meaning systems, as well as the analysts' interpretative resources (Braun & Clarke, 2023; Nelson & Prilleltensky, 2020). Service users' words, or descriptions of their experiences, provide access to their particular version of reality and research produces interpretations of this reality. Furthermore, this approach understands service users', and thus also co-researchers', reality as evolved through history and constituted by social and institutional structures within and surrounding health services. The researcher works in solidarity, then, with co-researchers, striving to amplify service users' voices through the research process.

## Developing a contextualist collaborative thematic approach

**Figure 1.**
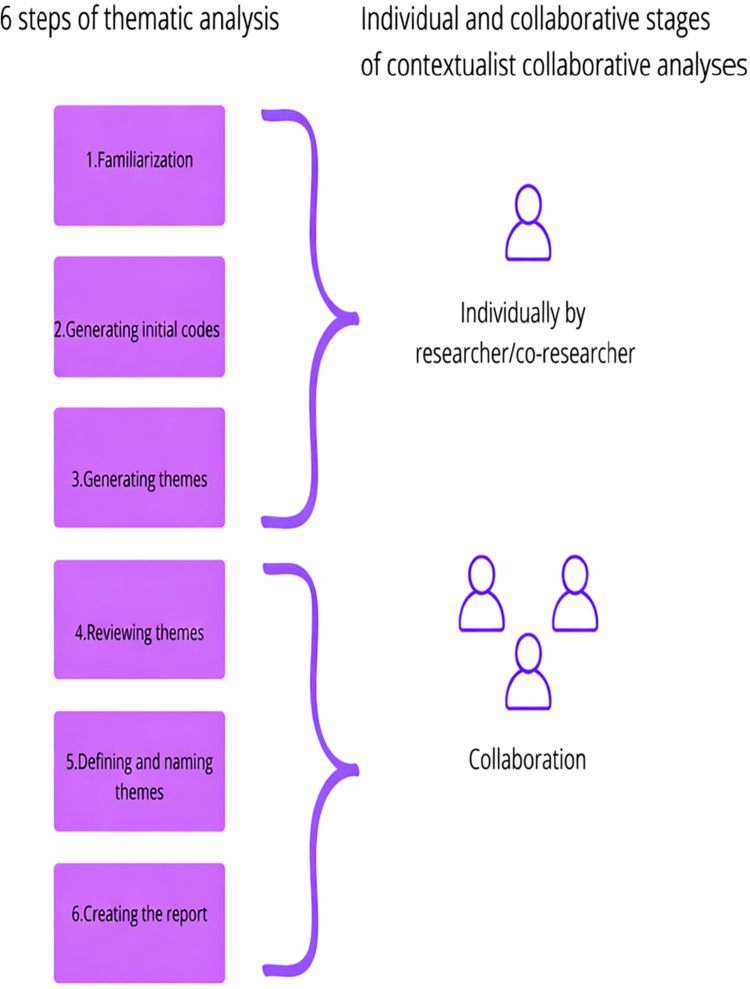
Individual and collaborative phases of contextualist collaborative TA.

Building on TA, as well as experiences from cultural-ideological analyzes (Bahl & Hagen, 2017; Bahl and Hagen, 2017; Bahl et al., 2021, 2025) and collaborative thematic analyzes with a co-researcher (Bahl et al., 2019; Bahl et al., 2022, 2023a, 2023b), we will now propose guidelines and examples for how to conduct individual and collaborative phases of contextualist collaborative TA with co-researchers (see Figure 1).

### Previous analytical work: cultural-ideological and collaborative analyses

In previous analyzes we have investigated phenomena such as multiple psychological sense of community (MPSOC), life-stages, culture, well-being, mattering, and recovery among different populations and age groups in different socio-cultural contexts (Bahl & Hagen, 2017; Bahl et al., 2017, 2019, 2021, 2022, 2023a, 2023b, 2025).

The cultural-ideological analyzes analyzed interviews from four samples (2 × 2 design): older adults and young adults in urban Norway and India. These cultural-ideological analyzes utilized three steps of analyzes, where the initial steps of TA were conducted, and then themes were analyzed with respect to two different macro-level meaning systems (urban India and Norway). Finally, the two age groups' individual meaning systems were compared by a comparative approach.

The collaborative thematic analyzes analyzed interviews from a Norwegian national study on service user experiences (Bahl et al., 2019, 2022, 2023a, 2023b) among different sub-groups having substance use problems. These analyzes included two researchers and one co-researcher which conducted collaborative thematic analyzes consisting of seven stages: 1) Coding and formulating initial themes by researcher one, 2) Coding and formulating initial themes by co-researcher, 3) collaborative revision of themes by researcher one and co-researcher, 4) Coding and formulating initial themes by researcher two 5) collaborative revision of themes by researcher one and two, 6) collaborative finalizing of themes by all analysts, 7) writing up the findings with authors (see Figure 2 for an example of how themes developed through these steps). As exemplified in Figure 2, our previous collaborative analyzes were inherently iterative, with themes emerging through an ongoing process of refinement and integration between analysts. The development of these stages for collaborative thematic analyzes, as well as the cultural-ideological frameworks for context-sensitive analyzes, were fundamental elements for the development of the proposed analytical approach, leading to more condensed stages of analysis and sensitization to context within these.

Figure 2.Example of a theme development process in collaborative thematic analysis.The figure illustrates how themes were refined and integrated in a previous collaborative thematic analys between three analyts.The figure illustrates an example of a theme development process from a previous collaborative thematic analysis. The process involved seve steps of analysis, where themes were refined and integrated between three analysts.

**Figure:**
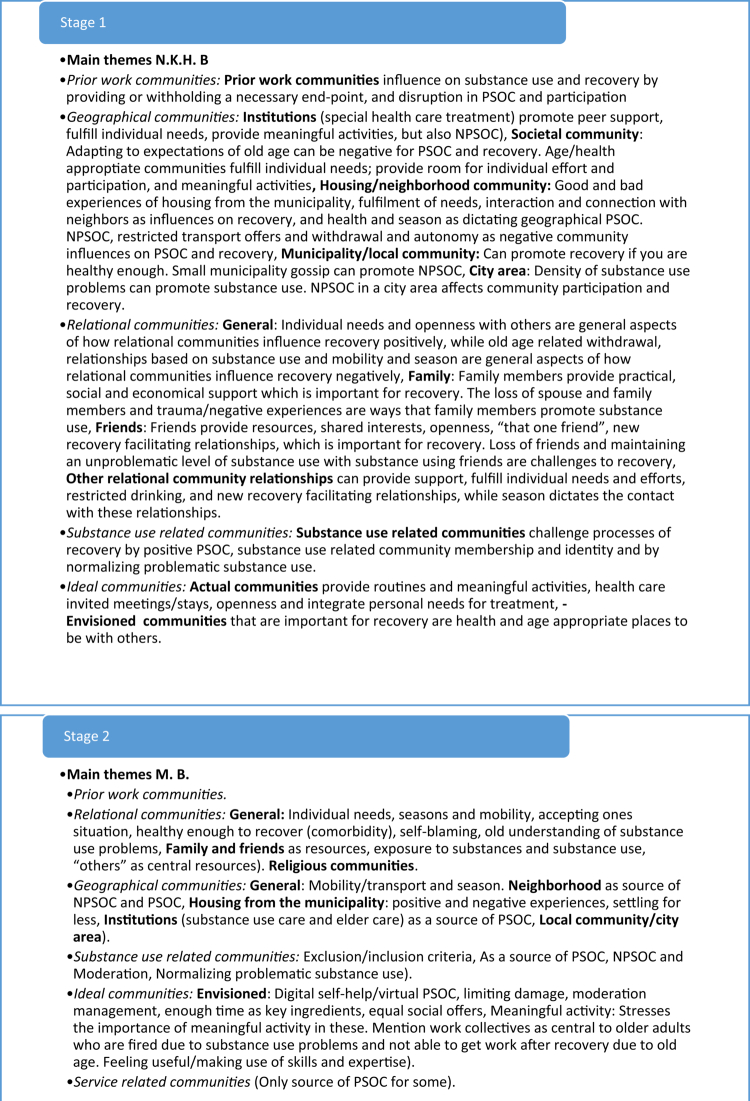

**Figure:**
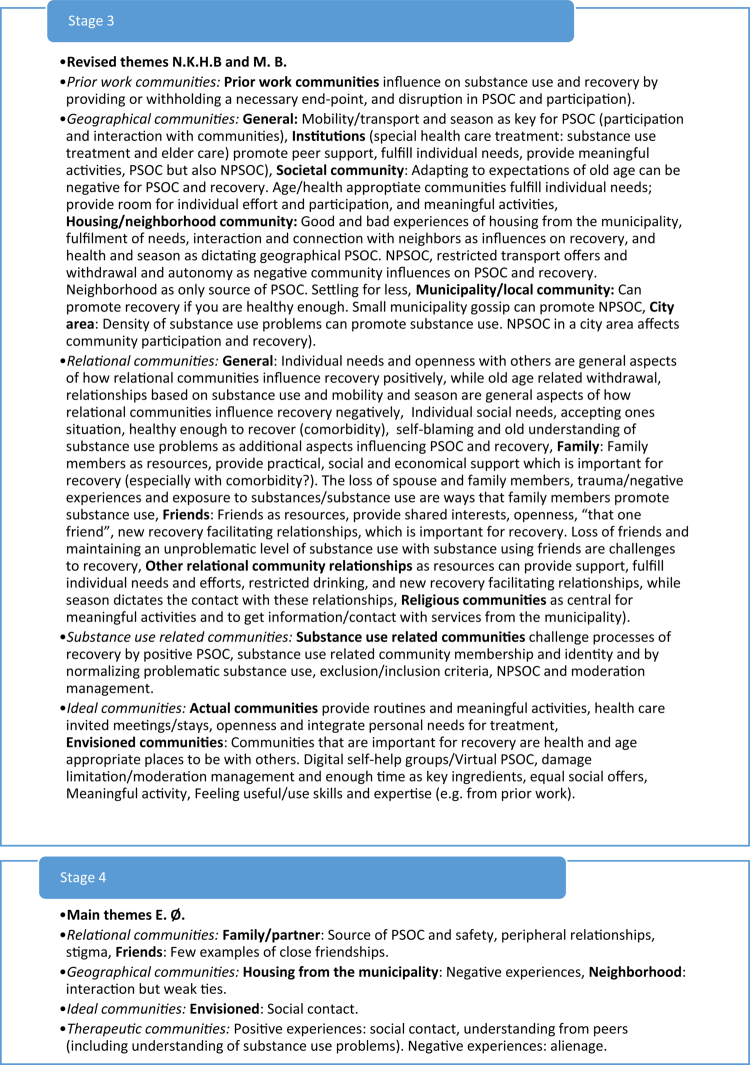

**Figure:**
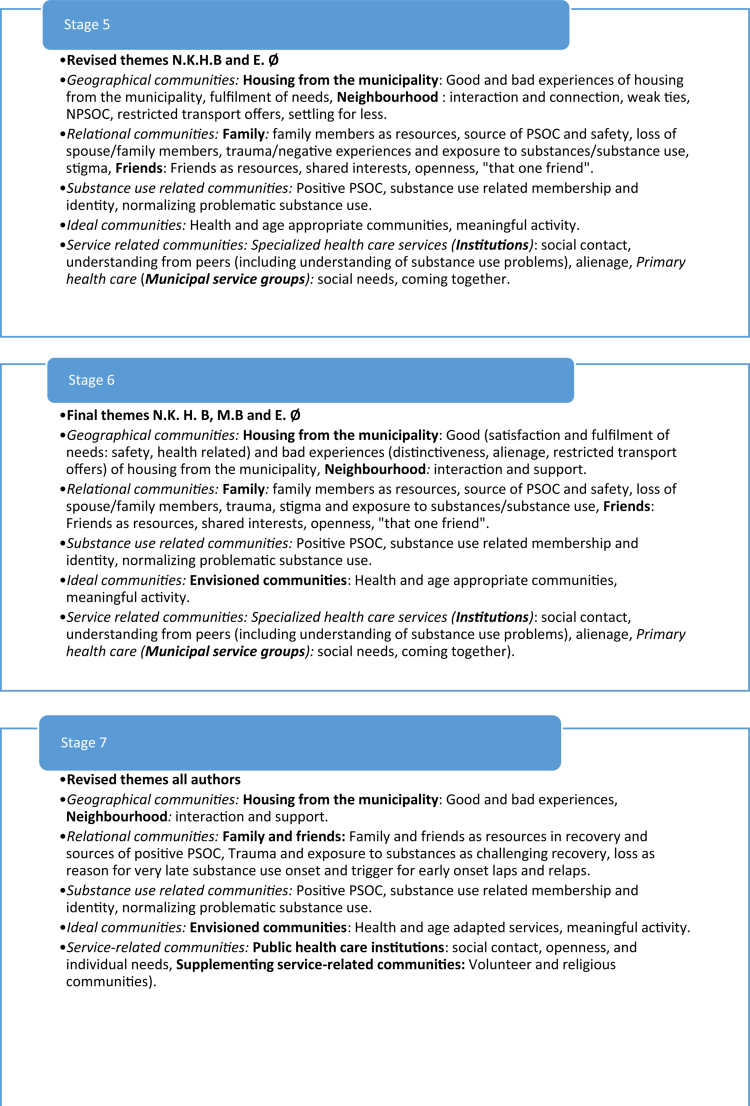

### Proposed analytical approach: four stages of contextualist collaborative TA

**Figure 3.**
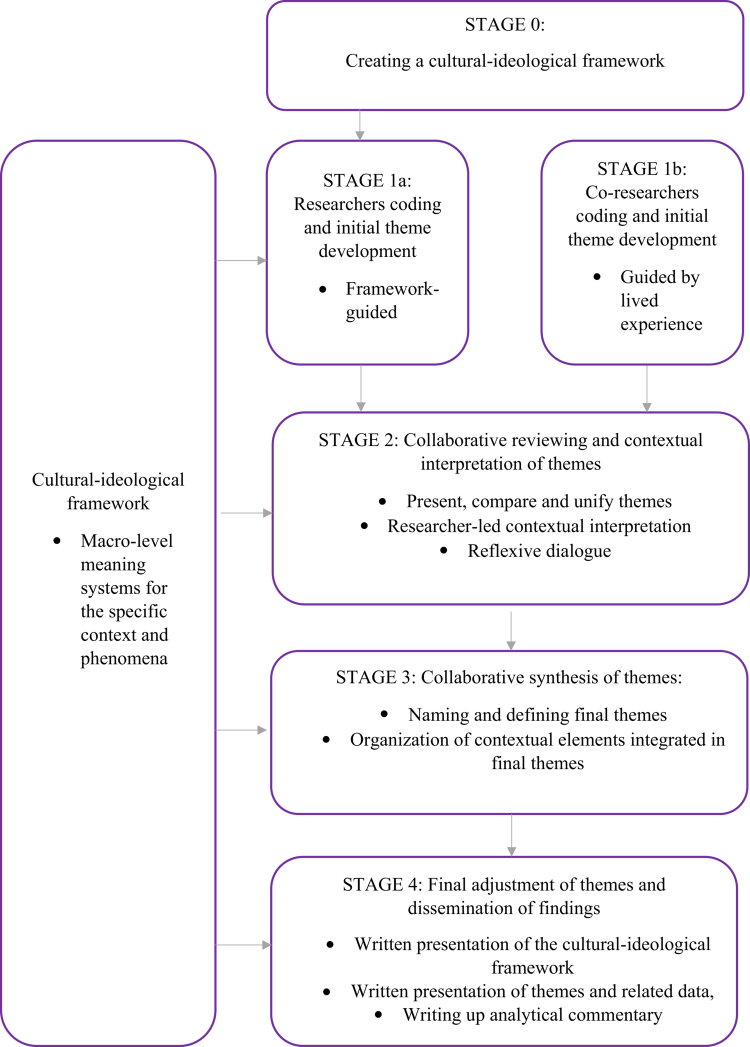
Iterative stages of contextualist collaborative TA.

To prepare the contextualist collaborative TA, the researcher creates a theoretical framework for the cultural-ideological context of the phenomena analyzed (see Figure 3). In our cultural-ideological analyzes we have drawn upon principles from indigenous psychology (Kim & Berry, 1993) and created cultural-theoretical frameworks for each phenomenon studied. This has been performed by synthesizing texts that articulate the meaning system of, for example, the MPSOC phenomena, within different socio-cultural contexts. In line with emic analytical principles, this work entails an extended review to include scholarly literature (books and journal articles) as well as culturally significant materials, such as religious writings, to ensure that the frameworks integrate contextually grounded and culturally embedded understandings.

1) Coding and development of initial themes

When the cultural-ideological framework is ready to apply, the researchers' can, as we have, utilize it in coding and initial theme development. Concretely, the framework is used to identify and interpret latent meanings lying underneath the data surface of what is taken for granted and expected by the participants. As such, the researchers' coding and initial theme development goes beyond description to interpret meanings (or health service experiences) as situated and is inherently deductive: viewing the data through a cultural-ideological lens.

Furthermore, a novel element for the suggested approach is that it assumes co-researchers' micro-level meaning systems as embedded within their cultural-ideological context. Thus, co-researchers code and develop initial themes inductively according to their perspective, before the researcher then interprets these according to the cultural-ideological framework (this interpretation is undertaken as part of the second stage of the analysis). This interpretative part of the approach has not been utilized in previous analyzes, and was developed specifically to further develop the approach from a collaborative analytical approach to a *contextualist* collaborative approach.

In our previous collaborative analyzes, the researchers have coded and developed initial themes parallel with the co-researcher, so that all analysts' codes and initial themes were ready before reviewing them. As such, this first stage involves the co-researcher although the co-creation takes place in the next step.

Finally, although we have analyzed interview data, we encourage researchers and co-researchers to utilize the approach in coding and systematizing all types of qualitative data of service user-experiences. We also advice analysts to document all analytical sessions. In example, as we have, one may write summarizing notes or minutes for each stage of the analysis as well as a log for the analytical process.

2) Collaborative reviewing and contextual interpretation of themes

The second stage of the approach involves reviewing and unifying the “first draft” of themes. This part of the analysis is where collaboration and co-construction is initiated. In our previous collaborative analyzes, three involved analysts have presented their initial themes—two by two in analytical sessions: one between the first author/researcher and co-researcher, and one between the two researchers. As such, in the first session, the first author presented her themes to the co-researcher and vice versa. The first author then noted the co-researchers' themes as part of the minute of the session (see Appendix for an example), which she then worked with after the session in unifying the two sets of themes. This work involved looking for overlaps between the two sets of themes and identifying nuances which could give a broad and deep understanding of the phenomena in focus.

Thus, the first author/researcher has taken the lead in guiding the revision and unification process. However, this role does not entail unilateral authority. After formulating a proposal for unifying themes, the first author goes back to the co-researcher to discuss these. In these discussions, the analysts go back and forth between the initial and merged themes until they reach an agreement. For instance, in one analysis (X) the co-researcher' articulated community transitions as non-linear; communities influence may shift in different phases of the recovery process. This experiential element added a more fluid, processual understanding of the participants' recovery. Additionally, the co-researchers' emphasized the need for recovering individuals to distance themselves from substance use-related communities, which confirmed the researchers' analysis, but added an experiential dimension to the sub-theme “adaptive negative psychological sense of community”.

Across our analyzes, the co-researcher contributed by introducing independent codes, sub-themes, and themes; confirming researcher-generated interpretations; and, critically, nuancing and re-orienting themes through experiential insights that deepened the understanding the participants' experiences. However, in cases where the researcher and co-researcher disagreed about codes and themes to include, both parts have presented their arguments and used validity criteria as guiding principles (see Yardley, 2015). Central guiding questions for these processes are: Do the sub-themes or themes provide breadth in the analysis? Do they provide depth or breadth in interpretations? Do they help us understand the participant's contexts better? And, do they influence the relevance of the impact and importance of the findings? Then, the same processes were repeated between the first author and the second researcher.

However, this stage doesn't end there. To integrate context-sensitivity, we propose that the researcher asks if and in what ways the specific elements within the cultural-ideological framework are reflected in the co-researchers' codes and themes. In example, in a previous analysis the first author generated a theme “Helping and caring for others” in urban Indian older adults' descriptions of PSOC. To further analyze this theme in a context-sensitive way, we used several elements from the cultural ideological framework of meaning systems in Mumbai. For instance, the concept “seva”; “service to others” (Jacobsen, 2014), which often understood as a selfless act of worship for a Guru or a social cause (Pandya, 2014; Srinivas, 2008). This helped us understand this theme; that the community efforts which the participants described, reflected a practice of seva, which today is frequently practiced by volunteering in NGOs (Bornstein, 2012). Central questions for integrating this kind of context-sensitivity broadly are: “How do the themes reflect elements within the cultural-ideological context, and how do the cultural-ideological elements help us in understanding the themes?”

Finally, to create a reflexive process about contextual aspects in the analysis, it is important that the researchers share and discuss their contextual interpretation of codes and themes with the other analyst. Central guiding questions are “how are contextual elements represented in this interpretation, and what do I take for granted by interpreting this as such?”

3) Collaborative synthesis of themes

Once the draft of unified themes is ready and accepted, all the analysts involved meet again, to carry out the third stage of the analysis; naming and defining final themes collaboratively. In this stage, the analysts work together to finalize the united themes; what should be the definition of the unified themes and what can be good names for capturing the essence of each theme. In previous analytical sessions for this stage, the main discussions have concerned which themes to move forward with for the articles. The first author recorded meetings by Zoom and audio, so that it was possible to go back and check what was said in the analytical sessions, including arguments and decisions made. This assisted the iterative collaborative analytical sessions as part of this stage.

Finally, for a contextualist approach, there also needs to be discussions about what contextual aspects that should be included in theme definitions and names of the final themes. As such, this stage involves a process of co-creation, a more narrow and tailored organization of contextual elements for each theme. In previous work the first author has gone back and forth between theme names and definitions and the cultural-ideological framework to evaluate which elements that are most crucial to proceed with for answering the research question(s). As such, a core agenda for this stage is to establish the final sensitization to the context of the findings.

4) Final adjustment of themes and dissemination of findings

In the fourth stage of the analysis, the final themes are adjusted—this time between all the authors, as part of the writing process. In previous work the focus for this stage, has been on conveying what was undertaken in the analysis, presenting the cultural-ideological framework, data excerpts and analytical commentary. In previous work, we have included a presentation of the cultural-ideological frameworks as a short written description of the context we studied, e.g. “The Indian context and older adults”. As such, the final touches of the analysis is a collaboration between authors of the article, adjusting dissemination of interpretations and layers included in the analysis.

To further continue the involvement of the co-researcher in our collaborative analyzes, we also have included the co-researcher among the authors. Thus, the co-researcher has been included in communications between authors in the writing process, providing input on manuscript drafts, disseminating the findings in popular scientific abstracts (see Bahl et al., 2025 for an example) and media interviews.

## Ethical statement

This method's article draws on experiences from several analyzes from two studies. Both studies were conducted in accordance with the Declaration of Helsinki. The first study (see Bahl & Hagen, 2017; Bahl et al., 2017; Bahl et al., 2021) was approved by NSD (Norwegian ethics comity). The second study (see Bahl et al., 2023a) was approved by the Data Protection Officer at St. Olavs Hospital in Trondheim, Norway (Reference ID: ESA 17/4211). All participants were informed about the study and gave written consent prior to their participation.

## Results

### Demonstrating validity in contextualist collaborative TA

We have argued, that contextualist collaborative TA with co-researchers, may ultimately lead to enhanced context-sensitive understandings within health service inquiries. We will now take a step further to demonstrate how the proposed approach aligns with, and may result in ensuring additional established validity dimensions for qualitative research: (1) sensitivity to context, (2) commitment and rigour, (3) transparency and coherence, and (4) impact and importance (Yardley, 2000, 2015).

Sensitivity to context: This dimension highlights the importance of considering the participants' context—here both the health care system and the surrounding sociocultural and ideological environment—to gain a valid and relevant understanding of service user experiences. By grounding researchers and co-researchers' codes and themes in a tailored cultural-ideological framework, the proposed analytical approach is designed to foreground sensitivity to context throughout the analysis; from coding to writing up the results (see Figure 3).

Commitment and rigour: This dimension relates to whether the analysis has been conducted with satisfactory breadth and depth (Yardley, 2015). With co-researchers' user-perspectives and a cultural-ideological framework integrated in codes and themes, the analysis can become both broader and deeper: adding broad and deep experiential and contextual dimensions to the analysis. One can, of course, provide depth and breadth to individual analyzes by including other researchers. However, one can only add situated experiential nuances by including user-perspectives in analyzes. As such, with the proposed systematic procedure for integrating these nuances throughout the analysis, researchers can be better equipped for generating committed and rigorous understandings of service users service experiences.

Transparency and coherence: Contextualist collaborative TA, like other qualitative approaches, applies several relevant tools which can aiding the transparency of analyzes: log writing, memos and use of audio recordings. However, what is particular to this approach, is that the collaboration with the co-researcher proposes the opportunity to enhance transparency in a manner sensitive to service users and their next of kin, by their assistance in formulating and sharing what was actually undertaken in popular scientific disseminations for these groups. Furthermore, integrating the user perspective in the analysis and dissemination can also assist the research team in retaining a coherent fit between research questions and interpretations relevant to health services.

Impact and importance: This dimension of validity emphasizes the significance and impact of the findings. As argued, co-researchers have user experience and a mindset, making them prone to contemplate about the analysis and findings in an applied manner with respect to the end-user. By integrating contextual and experiential dimensions in analyzes of user experiences, future health services research, thereby, can be able to generate enhanced context-sensitive and user-sensitive understandings, hopefully with consequences of more effective impact and relevance for health services.

## Discussion and concluding remarks

This methods article contributes to the scholarship on user-involved qualitative health services research by offering proposals for a systematic, context-sensitive framework for collaborative thematic analysis with co-researchers. While previous work has emphasized the value of user involvement in health services research (Boote et al., 2002; Moltu et al., 2013; Shippee et al., 2015; Thurston et al., 2023) and also proposed how to conduct collaborative qualitative analyzes with co-researchers (Eggebø, 2020; Pettersen et al., 2019), methodological guidance on how to co-create context-sensitive analyzes with co-researchers remain underdeveloped. By offering suggestions for structured, step-by-step guidelines for integrating multi-level contextual interpretations as well as co-researchers' perspectives with a familiar and accessible thematic analytical approach, our approach can thereby offer health services research potential benefits for enhanced context-sensitivity, and thus, more valid analyzes of service users situated experiences.

The above proposal is particularly significant given the ongoing paradigm shift in health services research, which increasingly values pluralistic knowledge systems and participatory approaches (Levitt et al., 2025; Wisdom et al., 2012). By rooting the proposed approach in a a) cultural-ideological framework (Bronfenbrenner, 1994; Levitt et al., 2025; Nafstad & Blakar, 2012; Valentim, 2011); b) contextualist epistemology (Clark et al., 2021; Nelson & Prilleltensky, 2020); and c) experiences from cultural-ideological (Bahl & Hagen, 2017; Bahl et al., 2017, 2021, 2025) and collaborative analyzes (Bahl et al., 2019, 2022, 2023a, 2023b), we move beyond positivistic and generic calls for user-involvement to integrate lived experiences as an analytical resource to context-sensitive research. Furthermore, the methodological rigour of our approach is strengthened by its alignment with dimensions of validity; demonstrating how contextualist collaborative TA can enhance context-sensitivity, commitment to rigour, transparency and coherence, as well as impact and importance.

To further develop this cultural-ideological approach, future studies should explore its application across diverse qualitative data and health care settings, to refine best practices for effective co-researcher engagement in collaborative health services research inquiries across different sub-fields.

### Implications for practice: strengths and limitations

The proposed approach presents several practical challenges as well as central promises for health services research as a practice. When it comes to challenges, the approach, most likely, requires additional time, resources, skills and complex commitments from researchers and co-researchers. For researchers, these commitments may include interpreting codes and themes from multiple perspectives, evaluating the influence of diverse contributions on the validity of findings, fostering, managing and conveying reflexive processes and bridging communication across the social worlds of researchers and co-researchers. For co-researchers, commitments to collaborative analysis may involve a significant additional workload parallel to other positions and demanding efforts in analytical sessions and reflexive practices while going through recovery.

Furthermore, although the proposed approach views the co-researcher's subjectivity and perspective as a resource, applying the approach with only one co-researcher carry inherent risks of isolation or reluctance to share input. In our previous collaborative analyzes, working with one single co-researcher, which was a reoccurring theme in the planning of our analyzes (Bahl et al., 2019, 2022, 2023a, 2023b). Although we attempted to include multiple co-researchers; however, the invited co-researchers did unfortunately not have capacity for additional work. This is a typical issue within user-involved research: Co-researchers are scarce, often involved in several research projects, in addition to holding other positions. As such, our analyzes involved a numerical imbalance, where the co-researcher worked alongside two researchers, raising concerns about potential power asymmetries. To mitigate these circumstances, the first author actively sought to ensure the co-researcher's inclusion in defining final themes by (a) repeatedly inviting him to share reflections on presented themes and sub-themes, (b) actively involving him in decisions about merging themes, and (c) and making sure that he was informed about the arguments for dismissing or adding themes. Despite these efforts, such analytical processes still carry inherent risks of imbalance and reluctance to share input. We therefore recommend that researchers strive to include multiple co-researchers or adopt additional strategies (e.g. meeting physically and informally to develop the needed alliance, documentation of interpretative decisions, iterative reflexive dialogue, and acknowledgement of analytical limitations in reporting) to address these dynamics and safeguard transparency of collaboration in analyzes.

Nonetheless, contextualist collaborative analyzes with co-researchers have the potential to generate context-sensitive, and thus also, more valid understandings of situated health service experiences. By utilizing co-researchers' perspectives and meaning systems, as “culture-ideological keys” in the steps of the proposed approach, health services researchers may be better equipped in obtaining and enhancing context-sensitive understandings of health service experiences across different cultural-ideological contexts.

To conclude, we hope to inspire a new line of qualitative health services inquiry: one that aligns with ongoing methodological shifts, systematically engages co-researchers in the co-construction of knowledge, and acknowledging service users and co-researchers as contextual beings.

## Data Availability

Documents from the analyzes which the article builds on are available upon request from the corresponding author. The full data sets are, however, not available due to ethical and privacy concerns for the participants.

## Endnotes

^1^
Similarly to service users, co-researchers have lived or are living experience as a patient or health services user for an illness. However, co-researchers also have training in collaborating with researchers throughout the research process.

^2^
Context-sensitivity refers to attentiveness to contextual distinctiveness and diversity (here, both with respect to the health care system and the surrounding sociocultural and ideological environment), which provides a critical foundation for the development of appropriate, culturally grounded, and community-responsive understandings and interventions (Maton, 2004; Nelson & Prilleltensky, 2020).

^3^
Meaning systems are shared frameworks that organize concepts within a socio-cultural context. These systems operate at multiple levels: macro (public discourse, institutional language, ideologies) and micro (individual word use). Furthermore, the levels are interrelated—micro-level meanings can shape macro-level meanings and vice versa (Billig, 1997; Blakar, 1979; Carlquist et al., 2017; Formanowicz et al., 2016; Holtgraves, 2014; Nafstad et al., 2009; Pennebaker et al., 2003; Pennebaker, 2011; Rommetveit, 1992). In health services research, this perspective underscores how language (e.g. descriptions of health service experiences) reflects and shapes understandings of health and related concepts within specific socio-cultural contexts (macro-level), as well as the individuals within them (micro-level).

^4^
For further details on qualitative analyzes involving teams of co-researchers, see Pettersen et al., (2019).

## Appendix

Meeting minute: Codes, sub-themes and themes by the co-researcher (17.12.20).

The co-researcher presented his themes from his analysis in a Zoom meeting. Relational communities (family, partners and friends) were highlighted the most central communities in the participants' recovery, in addition to ideal communities, individual relationships related to service related communities and substance use related communities.

Relational communities were described as functioning as resources supporting the participants in the recovery, as promoting contact with services, as motivation for recovery, but also as promoting exposure of substances and substance use.

Also, similar to the collaborative analysis between the first and second author, geographical communities were not seen as important for recovery. As such, relational communities and ideal communities were seen as most the meaningful themes/deductive tools for the material, while geographical communities did not contribute significantly to the analysis. When asked about institutions and housing from the municipality as geographical communities, the co-researcher confirmed these as communities with a negative impact on recovery, as they exposed the participants to substances and substance use. In addition, he highlighted that these communities were not recommended for young adult persons with substance use problems, especially those with moderate problems, as these could expose people to heavier substances and new substance use related communities.

In terms of ideal communities, the co-researcher highlighted the importance of these for promoting recovery. Both ideal communities which the participants had (often communities where one could do meaningful activities with other members) and communities which the participants ideally should have had were addressed.

Substance use related communities were described as the point of departure in recovery processes. It was important for recovery to leave or distance oneself from these communities to recover. Distance, alienage and similar notions are important dimensions of negative psychological sense of community (NPSOC) and regarded as central with respect to substance use related communities and recovery.

Another similar notion from the co-researcher's analysis was the importance of individual relationships with professionals working in the services. These relationships were described as supporting, deep connections, and essential for the experience of the service-related communities but also for receiving help from the services. The co-researcher also nuanced this theme, by addressing two new aspects. First by expressing that the service user's discourses (having the right language and saying the right things) were important for the participant's possibilities of help and recovery. And, secondly, by specifying that workers in NAV could be regarded as gatekeepers with respect to which communities one could be part of and that the workers labelling of the service user (as “high”/using substances) could restrict the possibilities of community membership and services received. As such, the participants' relationship to their contact persons in NAV was described essential for community membership and recovery. Both of these notions can be seen as notions of power.

Community interactions in recovery: The co-researcher confirmed the importance of community interaction in recovery and the identified interactions from the first author's thematic analysis. However, this was something that he wanted to go into depth in a separate conversation.

Finally, community transitions in recovery was presented as a central theme in the material. Similar to the first collaborative analysis, these transitions were part of the recovery processes and related to the change of needs as one recovered. However, these processes of community transactions and recovery were described as non-linear, going back and forth and hand-in-hand. In example, becoming sick could be related to going back to the substance use related community. Also, recovery could mean that you needed to leave self-help communities and finding a new community fulfilling new needs.

Co-researchers' themes:

1) Relational communities (family, partners and friends as resources (support and contact with services), motivation for recovery, exposure to substances and substance use).
2) Ideal communities (Meaningful activities with others and ideal communities needed).
3) Substance use related communities (negative influence, point of departure, NPSOC).
4) Individual relationships with professionals in service related communities (support, deep connection, experience and help from services, and power).
5) Community interactions in recovery (Family and substance use related communities, family and service-related communities, services and substance use related communities, family and neighbourhood).
6) Community transitions in recovery processes (non-linear).
